# Effects of dietary palmitoleic acid on vascular function in aorta of diabetic mice

**DOI:** 10.1186/s12902-022-01018-2

**Published:** 2022-04-18

**Authors:** Yasuhiro Takenouchi, Yoshie Seki, Sachiko Shiba, Kazuo Ohtake, Koji Nobe, Keizo Kasono

**Affiliations:** 1 Department of Pharmacology, Kawasaki Medical School, 577 Matsushima, Kurashiki, Okayama 701-0192 Japan; 2 Laboratory of Physiology, Faculty of Pharmaceutical Sciences, Josai University, 1-1 Keyakidai, Sakado, Saitama 350-0295 Japan; 3 Division of Pharmacology, Department of Pharmacology, Toxicology Therapeutics, School of Pharmacy, Showa University, Shinagawa-ku, Tokyo, 142-8555 Japan

**Keywords:** Diabetes, Palmitoleic acid, Thoracic aorta, Vasoconstriction

## Abstract

**Background:**

Chronic hyperglycemia in diabetes causes atherosclerosis and progresses to diabetic macroangiopathy, and can lead to coronary heart disease, myocardial infarction and cerebrovascular disease. Palmitoleic acid (POA) is a product of endogenous lipogenesis and is present in fish and vegetable oil. In human and animal studies, POA is reported as a beneficial fatty acid related to insulin sensitivity and glucose tolerance. However, few studies have reported its effects on aortic function in diabetes. Here, we investigated the effects of POA administration on vascular function in KKAy mice, a model of type 2 diabetes.

**Methods:**

Male C57BL/6 J (control) and KKAy (experimental) mice at the age of 14 weeks were used in the present study. For each mouse strain, one group was fed with reference diet and a second group was fed POA-containing diet for 2 weeks. The vascular reactivities of prepared aortic rings were then measured in an organ bath to determine if POA administration changed vascular function in these mice.

**Results:**

KKAy mice treated with POA exhibited decreased plasma glucose levels compared with mice treated with reference diet. However, endothelium-dependent vasorelaxant responses to acetylcholine and protease-activated receptor 2 activating protein, which are attenuated in the aorta of KKAy mice compared to C57BL/6 J mice under a reference diet, were not affected by a 2-week POA treatment. In addition, assessment of vasoconstriction revealed that the phenylephrine-induced vasoconstrictive response was enhanced in KKAy mice compared to C57BL/6 J mice under a reference diet, but no effect was observed in KKAy mice fed a POA-containing diet. In contrast, there was an increase in vasoconstriction in C57BL/6 J mice fed the POA-containing diet compared to mice fed a reference diet. Furthermore, the vasoconstriction in aorta in both C57BL/6 J and KKAy mice fed a POA-containing diet were further enhanced under hyperglycemic conditions compared to normal glucose conditions in vitro. In the hyperinsulinemic, and hyperinsulinemic combined with hyperglycemic conditions, vasoconstriction was increased in KKAy mice fed with POA.

**Conclusion:**

These results suggest that POA intake enhances vasoconstriction under hyperglycemic and hyperinsulinemic conditions, which are characteristics of type 2 diabetes, and may contribute to increased vascular complications in diabetes.

## Background

Diabetes mellitus is a chronic metabolic disease that is characterized by elevated levels of blood glucose due to insulin deficiency or (and) insulin resistance. Hyperglycemia in diabetic patients causes vascular abnormalities and progresses to diabetic macroangiopathy, including coronary heart disease, myocardial infarction and cerebrovascular disease [1]. The vascular abnormalities of diabetes mellitus are characterized by endothelial dysfunction and enhanced vasocontractility [2]. Moreover, cardiovascular complications associated with diabetes represent an issue in clinical practice because they result in vascular occlusion and restenosis after surgical intervention [3]. Although the common therapy for diabetes is performed on the basis of mean glycemic control using HbA1c levels, the prevention of the onset of macroangiopathy or neuropathy is very important for maintaining patient health [4, 5]. Indeed, moderate hyperglycemia has been shown to play a decisive role in in-stent restenosis even in non-diabetic patients, demonstrating the importance of glycemic control [6]. Diet remedies, such as dietary restrictions, are important for glycemic control, but it is also necessary to monitor potential dietary composition. In particular, most studies have suggested that high fat intake may increase the risk of insulin resistance and cardiovascular disease [7, 8]. However, it has been reported that the type of fats consumed, rather than the total amount of fat intake, may have a greater effect on the components of the metabolic syndrome [9, 10].

It has been reported that polyunsaturated fatty acids, which are often present in fish and shellfish, have a favorable effect on many lifestyle-related diseases including diabetes [11]. We have also reported the beneficial effects of fish oil or omega-3 polyunsaturated fatty acids (ω-3 PUFA) in in vitro and in vivo studies using type 2 diabetes model animals [12–14]. ω-3 PUFA reduces the risk of cardiovascular disorders by lowering LDL cholesterol levels in serum and has a direct action on aortic endothelial cells [14, 15]. Many large-scale epidemiological studies have examined the health benefits of fish oil or ω-3 PUFA intake for the prevention of cardiovascular diseases [16, 17]. However, other clinical studies reported that treatment with ω-3 PUFA has no effect on cytokines and platelet function associated with atherosclerotic disease in patients with type 2 diabetes [18].

In recent years, the monounsaturated fatty acid palmitoleic acid (POA) has also been recognized as an adipocyte-derived lipid hormone (or lipokine) that allows adipose tissue to regulate systemic metabolism, supporting its physiological relevance [17, 19–23]. Circulating POA has three main sources: intake as a natural food, endogenous lipogenesis (cis isomer) and dietary whole-fat dairy products (trans isomer). Both POA isomers have been reported to be associated with lower metabolic risk [19–23]. POA in fish oil and vegetable oil is also expected to increase insulin sensitivity [19, 20]. We have reported that POA decreased total cholesterol levels in serum and total lipid levels in the liver of high fat diet-fed mice [24]. Although, many studies have reported biological functions for POA, the health benefits of POA intake have not been elucidated. In this study, we investigated the effects of POA intake on diabetes-induced vascular abnormalities using thoracic aortas removed from KKAy mice, a model of type 2 diabetes.

## Methods

### Reagents

Acetylcholine chloride (ACh), phenylephrine hydrochloride (Phe), and Ser-Leu-Ile-Gly-Lys-Val-amide (protease-activated receptor 2-activating protein; PAR2-AP) were purchased from Sigma-Aldrich (St Louis, MO, USA). Sodium nitroprusside dehydrate (SNP) was purchased from FUJIFILM Wako Pure Chemical (Osaka, Japan). cis-9-Hexadecenoic Acid (palmitoleic acid; POA) was purchased from Tokyo Chemistry Industry (Tokyo, Japan). All reagents were dissolved in saline and concentrations are expressed as the final molar concentration in the ex vivo organ bath experiments.

### Experimental design

Male KKAy mice, which spontaneously develop obesity and type 2 diabetes [25], and control C57BL/6 J mice were obtained from Tokyo Laboratory Animals Science (Tokyo, Japan) at 6 weeks of age and fed a standard pellet diet (CE2; CLEA, Tokyo, Japan) for 8 weeks. Mice were exposed to a 12-h light–dark cycle and maintained at a constant temperature of 22 ± 2 °C and humidity of 55 ± 10%. KKAy and C57BL/6 J mice were then divided into 2 groups, respectively. For each mouse strain, one group was fed with reference diet and the second group was fed with diet containing POA for 2 weeks. Each group was described as follows: control mice, C57BL/6 J; control POA-treated mice, C57BL/6 J-POA; experimental mice, KKAy; experimental POA-treated mice: KKAy-POA. The composition of the diet was prepared based on the AIN93G diet [26]. During the experiment, the composition of POA diet was prepared according to daily weight fluctuations and food consumption in each mouse. The general diet compositions are shown in Table 1. Mice were euthanized by decapitation under isoflurane anesthesia, and blood samples were collected in tubes using heparinized funnels. The animal experiments were approved by the Institutional Animal Care and Use Committee of Josai University.

**Table 1:** Composition of the experimental diets

Ingredients	Reference diet	POA-containing diet	
Corn starch (g)	39.7486	39.7486	
Casein (g)	20	20	
α-Corn starch (g)	13.2	13.2	
Sucrose (g)	10	10	
Soybean oil (g)	7	^KKAy^ 6.807	^C57BL/6J^ 6.773
Cellulose (g)	5	5	
Mineral mix (g)	3.5	3.5	
Vitamin mix (g)	1	1	
L-Cysteine (g)	0.3	0.3	
Choline bitartrate (g)	0.25	0.25	
tert-Butylhydroquinone (g)	0.0014	0.0014	
Palmitoleic acid (g)	0	^KKAy^ 0.193	^C57BL/6J^ 0.227
Total(g)	100	100	

### Collection of plasma and measurement of plasma parameters

The blood samples were centrifuged (200×*g* for 20 min at 4 °C), and the supernatant plasma stored at − 20 °C until being assayed. Plasma levels of glucose, triglyceride, HDL cholesterol, total cholesterol and non-esterified fatty acid (NEFA) were each determined using commercially available enzyme kits (FUJIFILM Wako Pure Chemical). Plasma insulin was measured by enzyme immunoassay (Shibayagi, Gunma, Japan).

### Measurement of isometric force

Aortic vascular function was measured as previously described [27–29]. Briefly, the thoracic aorta was quickly isolated and immersed in oxygenated, modified ice-cold bicarbonate-buffered physiologic salt solution (PSS; containing 137 mM NaCl, 4.73 mM KCl, 1.2 mM MgSO_4_, 0.025 mM EDTA, 1.2 mM KH_2_PO_4_, 2.5 mM CaCl_2_, and 11.1 mM glucose). Next, the artery was separated from the surrounding connective tissue and cut into rings of 2-3 mm length under a stereoscopic microscope and suspended in a well-oxygenated (95% O_2_, 5% CO_2_) bath containing 10 mL of PSS at 37 °C. For the vasorelaxation studies, the rings were constricted with an equieffective concentration of prostaglandin F_2α_ (PGF_2α_) (1 × 10^− 6^ − 3 × 10^− 6^ mol/L). When the PGF_2α_-induced contraction had reached a plateau level, ACh (10^− 9^ − 10^− 5^ mol/L), SNP (10^− 10^ − 10^− 5^ mol/L) or PAR2-AP (1 × 10^− 8^ − 3 × 10^− 6^ mol/L) was added in a cumulative manner. The evaluation of vasodilation was expressed as a percentage of the level observed prior to adding PGF_2α_. The tension generated by the aortic rings was amplified and digitized via a transducer (TB-611 T; Nihon Koden, Tokyo, Japan) and recorded and stored using Lab Chart software (ADInstruments, Tokyo, Japan). For the vasoconstriction studies, the aortic rings were contracted by cumulative administration of Phe. After isolating the aorta, as described above, and cut into rings of 1.5–2.0 mm length, the rings were placed in oxygenated PSS. Next, vascular rings were mounted horizontally onto the microvascular force measurement system. In this vasocontraction study, the normal physiological glucose concentration (11.1 mM) was defined as the normal glucose condition. To understand the direct effects of extracellular glucose accumulation, a high glucose condition was established by pretreating the vascular tissues with high glucose-PSS (22.2 mM glucose in PSS) at 37 °C for 30 min, as previously reported by our laboratory [29]. These values were based on reported postprandial glucose levels in C57BL/6 J (~ 11.1 mM) and KKAy mice (~ 22.2 mM) in vivo [30, 31]. The obtained change in vascular tension was shown as the generated tension per 1 mm length of the aortic ring.

### Statistical analysis

All results are expressed as mean ± standard error of mean (S.E.M.). Numbers of mice and samples analyzed are indicated in the figure legends and table. Plasma parameters and body weight were compared by analysis of variance (ANOVA) followed by Tukey’s multiple comparisons test. Differences in responses between groups of aortic rings were determined by comparing the whole concentration-response curves using a two-way repeated-measures ANOVA with Tukey multiple comparisons test. *P* < 0.05 was considered statistically significant. The statistical analyses were performed using Prism 8 software (GraphPad Software, San Diego, CA, USA).

## Results

### Body weight and food intake

**Fig. 1:**
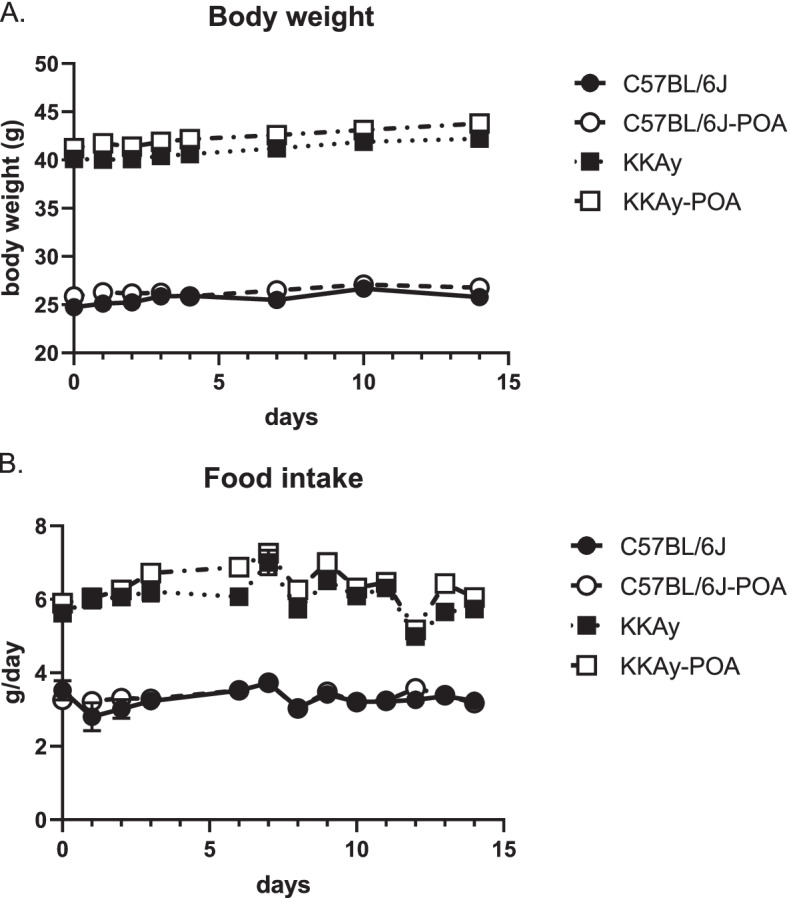
Body weight (**A**) and food intake (**B**) during the experimental period. The horizontal axis represents the number of days since the reference or POA-containing diet was fed to mice. Data are expressed as mean ± S.E.M. **A ***n* = 10 for all groups. **B ***n* = 3 for C57BL and C57BL-POA, *n* = 10 for KKAy and KKAy-POA

To determine the composition of the diet to be administered, we measured body weight and food intake after 8 weeks on a standard pellet diet. Body weights and food intakes were 25.1 ± 0.3 g, 3.3 ± 0.05 g/day (C57BL/6 J) and 39.9 ± 0.5 g, 6.2 ± 0.1 g/day (KKAy), respectively. We then measured body weight and food intake while mice were fed the reference or POA-containing diet. There was no difference in the body weight and food intake of the reference diet groups and POA-fed groups in both KKAy and C57BL/6 J mice (Fig. 1).

### Plasma parameters

**Fig. 2:**
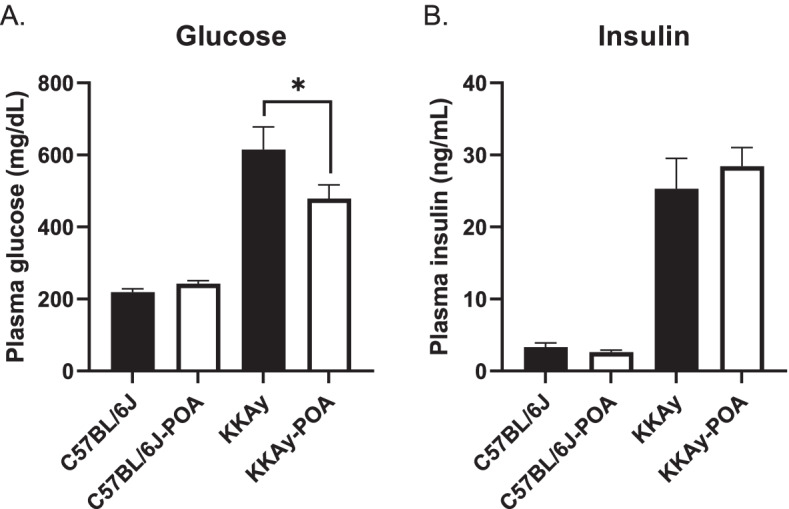
Plasma glucose (**A**) and insulin (**B**) levels in the four experimental groups. Data are expressed as mean ± S.E.M. of 7-11 mice. *P < 0.05, ***P < 0.001 (one-way ANOVA with Tukey’s multiple comparison test)

As shown in Fig. 2, POA administration had no effect on non-fasting plasma levels of glucose and insulin in C57BL/6 J mice. Administration of the POA-containing diet to KKAy mice for 2 weeks resulted in a significant decrease in plasma glucose levels (Fig. 2A), while plasma levels of insulin did not change compared to the reference diet group (Fig. 2B). In addition, HDL cholesterol levels in C57BL/6 J-POA showed a significant increase compared to C57BL/6 J (Table 2). There was no difference in the other plasma parameters (triglyceride, total cholesterol and NEFA) due to POA administration in both C57BL/6 J and KKAy mice (Table 2).

**Table 2:** Plasma levels of triglyceride, HDL cholesterol, total cholesterol and non-esterified fatty acid (NEFA)

Parameters	C57BL/6 J	C57BL/6 J-POA	KKAy	KKAy-POA
Triglyceride (mg/dL)	227.8 ± 31.1	206.0 ± 24.5	901.0 ± 227.6	955.1 ± 167.1
HDL cholesterol (mg/dL)	37.0 ± 4.5	59.1 ± 5.5 **	92.9 ± 13.1	85.1 ± 11.3
Total cholesterol (mg/dL)	146.0 ± 18.9	139.5 ± 10.5	174.0 ± 34.4	215.8 ± 20.4
NEFA (mEq/L)	0.37 ± 0.05	0.44 ± 0.04	0.65 ± 0.12	0.62 ± 0.09

Values are means ± S.E.M

**p < 0.01 vs C57BL/6 J mice

### Vascular reactivity

**Fig. 3:**
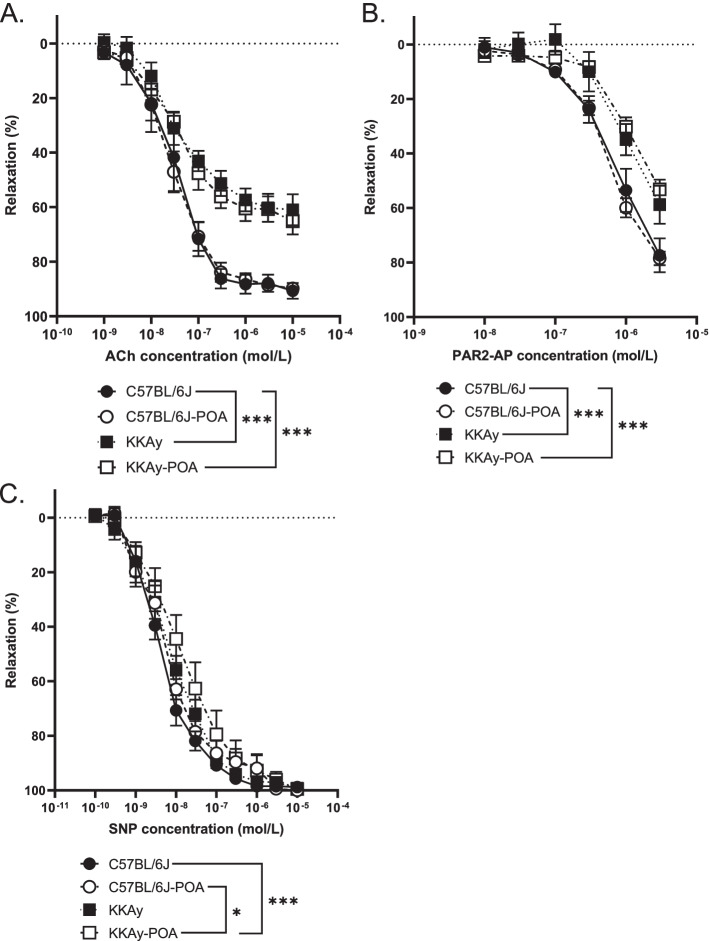
Concentration–response curves following ACh (**A**), PAR2-AP (**B**) and SNP (**C**) treatments of aortic rings. Data are expressed as mean ± S.E.M. of 6 or 7 mice. **P < 0.05, ***P < 0.001 (comparison of the whole concentration-response curves using two-way ANOVA with Tukey multiple comparison test)

After precontraction with PGF_2α_ (10^− 6^ − 3 × 10^− 6^ mol/L), cumulative treatment of ACh (10^− 9^ − 10^− 5^ mol/L), PAR2-AP (1 × 10^− 8^ − 3 × 10^− 6^ mol/L) or SNP (10^− 10^ − 10^− 5^ mol/L) was performed. The ACh- and PAR2-AP-induced relaxation of aortic rings isolated from KKAy mice were significantly weaker than those of C57BL/6 J mice administered the reference diet (Fig. 3A, B). The administration of POA for 2 weeks had no effect on the vasorelaxant response in both the KKAy and C57BL/6 J groups. The relaxation induced by the direct NO donor SNP showed no significant difference between C57BL/6 J and KKAy (Fig. 3C). However, the relaxation induced by SNP in aorta from KKAy mice administered POA-containing diet was attenuated compared with those from C57BL/6 J mice treated with reference and POA diet (Fig. 3C).

**Fig. 4:**
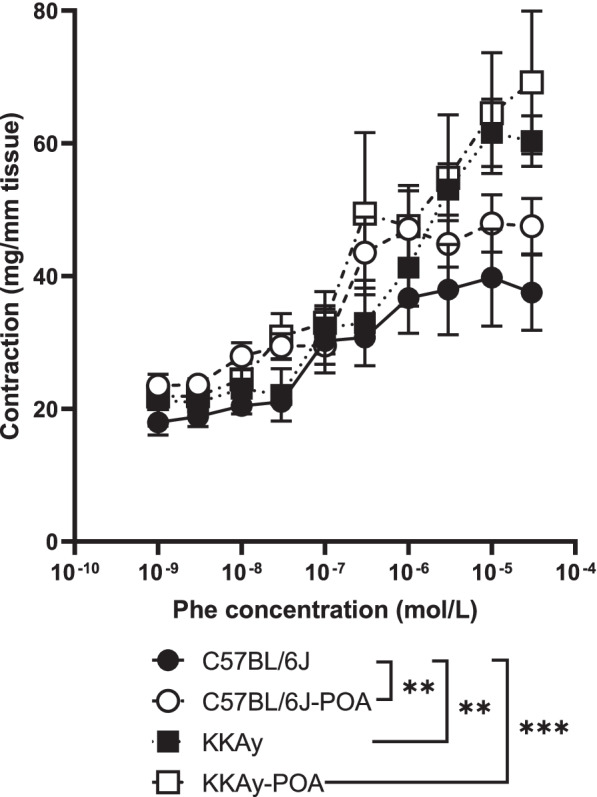
Concentration–response curves following Phe treatment of aortic rings. Data are expressed as mean ± S.E.M. of 4 mice. *P < 0.01, ***P < 0.001 (comparison of the whole concentration-response curves using two-way ANOVA with Tukey multiple comparison test)

**Fig. 5:**
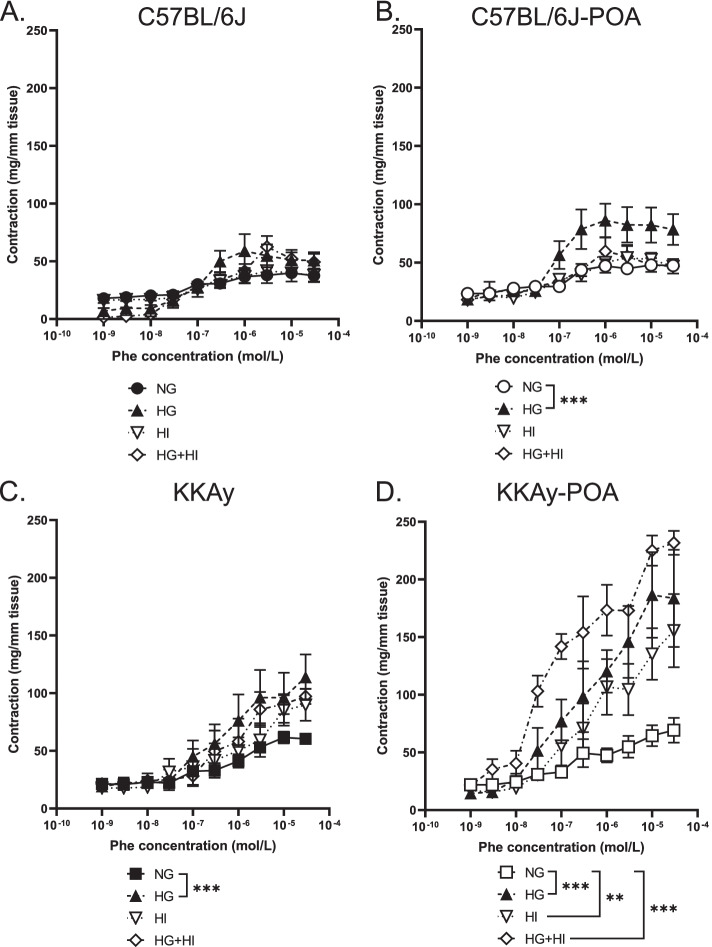
Concentration–response curves following Phe treatment of aortic rings. Organ baths contained normal glucose (NG: 11.1 mM), high glucose (HG: 22.2 mM) or high insulin (HI: 10 μM). Data are expressed as mean ± S.E.M. of 3-4 mice. *P < 0.01, ***P < 0.001 (comparison of the whole concentration-response curves using two-way ANOVA with Tukey multiple comparison test)

We then examined vascular contractile responses caused by Phe, which stimulates α_1_-adrenergic receptors (Figs. 4 and 5). Phe-induced vasocontraction in the aorta from KKAy mice was significantly greater than in C57BL/6 J mice. In the aorta from POA-administered mice, the vasoconstrictive response was enhanced in C57BL/6 J mice, but no change was observed in KKAy mice (Fig. 4). Next, the effect of direct treatment with extracellular glucose or/and insulin concentration on Phe-induced aortic contractile responses was examined (Fig. 5). The high glucose condition (HG) was performed by treating the tissue for 30 min in PSS containing 22.2 mmol/L glucose, which is double the normal glucose levels (NG; 11.1 mmol/L), a condition that models transient postprandial hyperglycemia. In high glucose-PSS-treated aorta isolated from C57BL/6 J mice, only a slight increase in contraction was observed when Phe was added at 3 × 10^− 7^ mol/L or higher (Fig. 5A). The aortas from the other groups showed high vasocontraction in HG compared with NG (Fig. 5B-D). Comparing Phe-induced vasoconstriction in the aorta from C57BL/6 J and KKAy mice under high glucose conditions, the aorta of POA-fed mice showed increased constriction compared to the aorta of mice fed a reference diet (Fig. 5A-D). Similarly, Phe stimulation was performed in the presence of 10 nmol/L insulin to model a condition that is comparable to postprandial hyperinsulinemia. In the high insulin condition (HI), the vasoconstriction responses of the aorta from C57BL/6 J, −POA and KKAy mice were not affected, but the response of the aorta from KKAy-POA mice showed a significant increase in tension (Fig. 5B-D). The Phe-induced vasoconstrictive response under high glucose and high insulin conditions also showed the same results as in the high insulin conditions (Fig. 5B-D).

## Discussion

The results of the present study showed that chronic dietary POA supplementation enhances vasoconstrictive responses under high glucose and insulin conditions in aorta from KKAy diabetic mice.

Changes in body weight and food intake were obtained during the 2 weeks of POA administration, as shown in Fig. 1; however, body weights remained largely unchanged during the treatment period for both the C57BL/6 J and KKAy mice. Although the exact amount of POA intake was not precisely controlled in our experiments, we estimated that the mice were fed POA at approximately 300 mg/kg/day based on their body weights, diet components, and food compositions. The dosage was chosen based on reports of improved glucose uptake at this dose [32, 33]. After the C57BL/6 J and KKAy mice were fed the experimental diets for 2 weeks, plasma parameters for all mice were measured. As shown in Fig. 2, insulin levels were not changed by POA treatment, whereas a decrease in glucose levels was observed in the plasma from KKAy mice. Our observations are consistent with reports that POA improves insulin sensitivity [19, 34].

Since it has been reported that endothelial-derived NO production decreases under diabetic conditions and vascular tissue becomes chronically insufficiently relaxed [35], we measured endothelium-dependent vasorelaxation. To measure endothelium-dependent vasorelaxation, we used ACh and PAR2-AP, which cause NO-mediated vasodilation in the artery [36, 37]. It has been reported that these vasorelaxant responses are impaired under diabetic conditions [38, 39]. Our results also showed that the endothelium-dependent vasodilation by ACh and PAR2-AP were attenuated in aorta from KKAy mice (Fig. 3A, B). There was no change in vasorelaxation in response to the NO donor SNP between C57BL/6 J and KKAy mice fed a reference diet, indicating that endothelial dysfunction occurred in KKAy mice. Because plasma glucose levels are significantly reduced following 2 weeks of POA administration in KKAy mice, we expected an improvement in endothelial function in KKAy-POA. However, endothelial dysfunction in KKAy mice did not improve following POA administration. Although we have reported on several substances that alter endothelial function in the absence of decreased plasma glucose levels [14, 28, 40], POA may not improve endothelial function despite its ability to decrease blood glucose levels. On the other hand, POA-induced changes were observed in the aortic contractile response (Figs. 4 and 5). The Phe-induced contractile response in aorta from KKAy mice reported in this study was enhanced compared with C57BL/6 J mice. It is thought that more pronounced vasoconstriction occurs in diabetic conditions due to an increase in contractile ability combined with a decrease in relaxation ability. Although POA treatment of KKAy diabetic mice for 2 weeks did not change the Phe-induced vasoconstrictive response, enhanced aortic contraction was observed in C57BL/6 J mice after POA administration (Fig. 4). Such elevated aortic contractility is expected to contribute to vascular disorders including coronary artery disease and stroke [41]. In addition, enhanced vasoconstriction in the aorta of C57BL/6 J -POA, KKAy and KKAy-POA mice resulted in further increases under high glucose conditions in vitro (Fig. 5B-D). In our experiments, 11.1 mM and 22.2 mM glucose were used as normal and high glucose conditions, respectively. When vascular tension is measured under high glucose conditions, it is known that arterial contractile responses are significantly increased in diabetes [42]. Activation of the Rho kinase pathway induces enhanced vasoconstrictive responses in vascular smooth muscle in diabetic model animals [43]. It has also been reported that activated PKC and ERK1/2 signals induce the proliferation of vascular smooth muscle cells leading to increased vasoconstrictive responses in high glucose conditions in vitro [44]. We observed the direct effects of insulin on ex vivo contractility and found that a high-insulin condition alone had no effect on vasoconstriction in aorta from C57BL/6 J and C57BL/6 J -POA (Fig. 5A, B) mice. In contrast, the Phe-induced vasoconstrictive response tended to be enhanced in the aorta of KKAy mice under hyperinsulinemia compared to the insulin-free condition of reference diet fed mice (*p* = 0.07), and was significantly enhanced in the POA diet fed mice (Fig. 5C, D). Although the effect of insulin on the response of vascular smooth muscle is not well understood, it is known that insulin treatment promotes the proliferation of vascular smooth muscle cells [45]. In our study, only 30 min insulin administration enhanced the contractile responses of aorta in KKAy and KKAy-POA mice. The vasoconstrictive response in both the high glucose and insulin conditions were similar to those of the high insulin condition alone (Fig. 5). Eicosanoids are biologically active lipid mediators derived from PUFA that are one of the factors thought to enhance vasoconstriction of aortic rings [46]. Changes in the vascular production of prostanoids, a major class of eicosanoids, have been reported in fructose-overloaded rats that show insulin resistance [47]. It is possible that there is an interaction between eicosanoids and insulin. This interaction may contribute to the difference in vascular contractile response between C57BL/6 J and insulin-resistant KKAy mice in the presence of insulin. POA administration significantly enhanced the aortic vasoconstrictive responses in both C57BL/6 J and KKAy mice under the high glucose condition (Fig. 5B, D). Therefore, it was suggested that POA induces enhanced vasoconstriction. POA is also known as a gap junction inhibitor, and it has been reported that inhibition of gap junctions causes a decrease in blood vessel diameter [48]. The increased vasoconstriction in the aorta of POA-fed mice is consistent with this report. Regarding other fatty acids, dietary saturated fatty acid has also been reported to enhance vasoconstriction [49]. On the other hand, it has been reported that PUFA biosynthesis in vascular smooth muscle cells is involved in calcium release associated with Phe-induced vasoconstriction [50]. In addition, dietary fish oil is rich in PUFA and inhibits vasoconstriction in association with reduced thromboxane A_2_, a vasoconstriction-inducing prostanoid [51]. These reports suggest that the circulating lipid balance, a product of fatty acid intake and biosynthesis, may alter vascular responses. In a clinical study, higher circulating POA in metabolic syndrome was found to be related to cardiometabolic risk [52]. The results of this report indicate that POA causes enhanced vasoconstriction, but further investigation is required to fully elucidate this effect.

## Conclusions

Although previous studies showed that POA treatment of diabetes mellitus has the beneficial effect of lowering blood glucose or lipid levels, it has also been shown to increase vasoconstriction, especially under the high glucose and high insulin conditions in this study. Thus, ingestion of high amounts of POA may contribute to increased vascular complications in poorly-controlled diabetes.

## Data Availability

All data generated or analyzed during this study are included in this published article.

## Abbreviations

ACh: Acetylcholine chloride
ANOVA: Analysis of variance
HG: High glucose condition
HI: High insulin condition
NEFA: Non-esterified fatty acid
NG: Normal glucose
PAR2-AP: Protease-activated receptor 2-activating protein
PGF_2α_: Prostaglandin F_2α_
POA: Palmitoleic acid
PSS: Physiologic salt solution
Phe: Phenylephrine
S.E.M.: Standard error of mean
SNP: Sodium nitroprusside dehydrate
ω-3 PUFA: Omega-3 polyunsaturated fatty acids
